# The Value of Contrast-Enhanced Ultrasound in the Evaluation of Carotid Web

**DOI:** 10.3389/fneur.2022.860979

**Published:** 2022-04-27

**Authors:** Qingqing Zhou, Rui Li, Shuo Feng, Fengling Qu, Chunrong Tao, Wei Hu, Yuyou Zhu, Xinfeng Liu

**Affiliations:** Stroke Center & Department of Neurology, The First Affiliated Hospital of USTC, Division of Life Sciences and Medicine, University of Science and Technology of China, Hefei, China

**Keywords:** carotid web, stroke, contrast-enhanced ultrasound, diagnosis, thrombus

## Abstract

**Objectives:**

The purpose of this study was to investigate whether contrast-enhanced ultrasound (CEUS) is more advantageous than conventional ultrasound in the diagnosis of carotid web (CaW) and to compare the clinical characteristics of patients in different age groups.

**Methods:**

Seventeen patients admitted to the hospital from October 2019 to December 2021 were included in our study. Patients were initially diagnosed with CaW using digital subtraction angiography (DSA), and conventional ultrasound and CEUS were completed. Baseline patient data were analyzed and compared between the <60 years old CaW group and the ≥60 years old CaW group to explore the differences between the two groups. Then, comparing the accuracy of conventional ultrasound and CEUS.

**Results:**

A total of 17 CaW patients participated in this study, including 4 female patients (23.5%) and 13 male patients (76.5%), with an average age of 59.41 (±10.86) years. There were 9 patients (52.9%) with left CaW and 8 patients (47.1%) with right CaW. Acute ischemic stroke (AIS) occurred in 14 patients (82.4%). Thrombosis occurred in five of 17 patients (29.4%). There was a significant statistical difference about the thrombosis between the <60 years old CaW group and the ≥60 years old CaW group [<60 years group: 0 (0%), ≥60 years group: 5 (62.5%), *P* = 0.005]. Seven patients (41.2%) received medical management, nine patients (52.9%) had carotid artery stenting (CAS), and one patient (5.9%) had carotid endarterectomy (CEA). None of the patients had recurrent stroke during the follow-up period. The diagnostic rate of CaW and thrombus by CEUS was higher than that by conventional ultrasound, and there was a significant statistical difference in the diagnosis of thrombus between CEUS and conventional ultrasound (χ2 = 4.286, *P* = 0.038).

**Conclusions:**

CEUS may have a higher diagnostic accuracy for CaW with thrombosis, and it has a higher clinical application prospect.

## Introduction

Stroke is a disease with high morbidity and disability rates (1) and is the second leading cause of death worldwide (2). Carotid web (CaW) can be detected in nearly 5% of young cryptogenic stroke patients (3). Therefore, CaW is a risk factor in cryptogenic stroke (4). CaW is a kind of endovascular variation caused by fibromuscular dysplasia (FMD) (5). It usually originates from the posterior wall of the carotid bulb and protrudes into the lumen, presenting a shelf-like structure in morphology (6). FMD was first proposed by Connett and Lansche (7), and the term “web” was first described in the literature by Momose and New (8). CaW is commonly associated with the occurrence of an ipsilateral cerebral infarction or transient ischemic attack (TIA) (9), suggesting its involvement in potential stroke mechanisms (3). CaW is prevalent in 1–7% of the population (10) and is more common in younger patients with cryptogenic stroke (3).

Although there are definite morphological characteristics of CaW, the diagnosis of CaW is clinically difficult and easily misdiagnosed or missed (11) because of its rarity, small size, and insufficient understanding (12). Conventional ultrasound is a non-invasive, rapid, and convenient method. It can be repeated many times and has a wide range of applicability (13). Contrast-enhanced ultrasound (CEUS) is a non-invasive method developed using conventional ultrasound technology as a foundation that provides more information, better image quality, and more quantitative data through intravenous injection of contrast agent as tracer (13). CEUS can clearly show conditions in the lumen, such as whether there is a contrast filling defect, and evaluate the degree of thrombus formation in the lumen. Therefore, CEUS is often used to display neovascularization in plaques in both symptomatic and asymptomatic patients, identify plaque vulnerability, and find the location of the thrombus and the site of involvement (14). CEUS is a more accurate method to measure the stenosis of lumen than conventional angiography and magnetic resonance angiography (MRA) (15).

Previous studies have explored the diagnosis of CaW using computed tomographic angiogram (CTA), high-resolution magnetic resonance imaging (MRI), and conventional ultrasound (16). However, few studies have explored the diagnostic value of CEUS for CaW. In particular, the diagnosis of superimposed thrombosis by CEUS has not yet been reported. Therefore, there is some confusion about the application value of CEUS in CaW and thrombosis. In addition, the incidence and severity of stroke is age-related and increases with age (17). The elderly patients with acute ischemic stroke (AIS) showed specific characteristics and epidemiology, with more baseline comorbidities, disability and higher NIHSS score at admission compared with younger patients (18). Moreover, the rate of thrombosis increases with age (19). To our knowledge, no studies have reported the characteristics of CaW in different age groups. Therefore, the purpose of this study was to identify different manifestations of CaW and thrombosis using CEUS and to explore the diagnostic value of CEUS. In addition, comparing the clinical characteristics of patients with CaWs in different age groups.

## Methods

### Study Population and Design

Patients diagnosed with CaW at the First Affiliated Hospital of the University of Science and Technology of China from October 2019 to December 2021 were retrospectively analyzed. The inclusion criteria were as follows: (1) age older than 18 years; (2) digital subtraction angiography (DSA) used to diagnose CaW; (3) improved conventional ultrasound and CEUS examination. The exclusion criteria were as follows: (1) incomplete medical history; (2) severe infection, tumor, failure of the liver, kidney, or respiratory system; (3) no follow-up data. Baseline characteristics and associated risk factors were collected, including sex, age, hypertension, diabetes, hyperlipidemia, coronary heart disease, history of stroke, alcohol, and smoking. In addition, the low-density lipoprotein (LDL), high-density lipoprotein (HDL), glucose, total cholesterol (TC), and triglyceride (TG) levels of patients were also recorded. Moreover, the length, width, location of CaWs, and degree of carotid artery stenosis were measured.

### Imaging Assessment and Analysis

**Figure 1:**
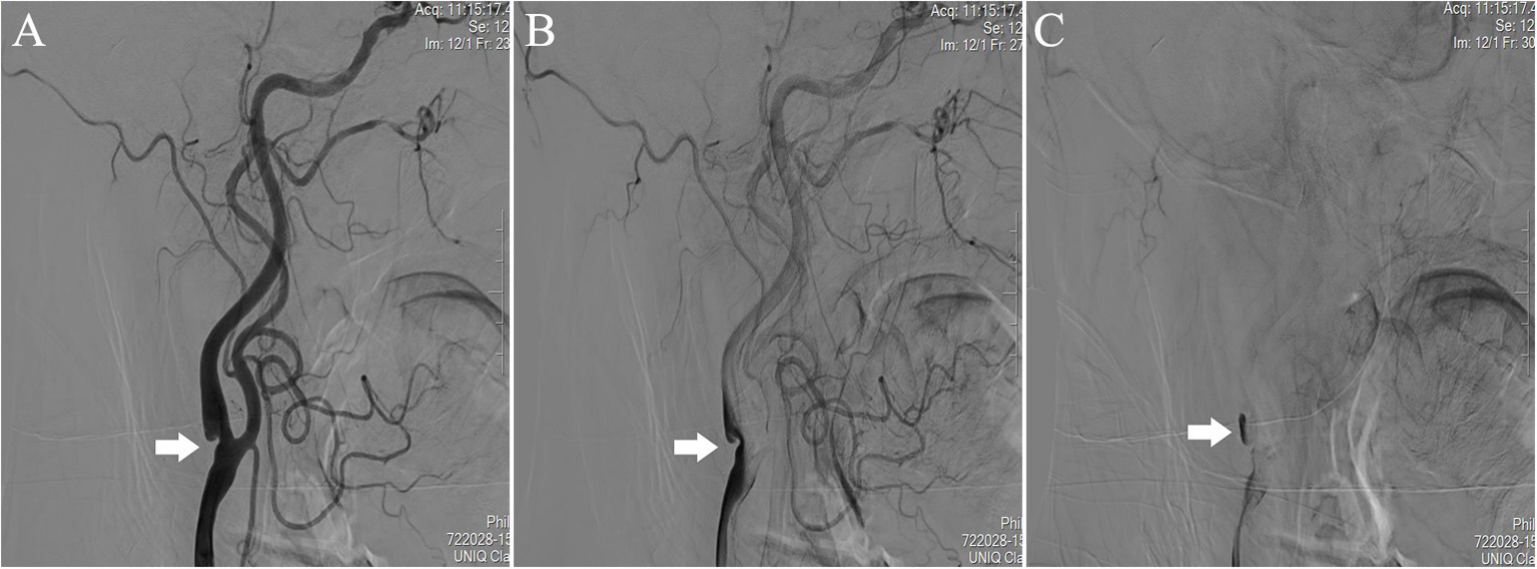
Performance of CaW at the right internal carotid artery of the patient during DSA. Comparied with **(A)** early-arterial phase, **(B)** mid-arterial phase, and **(C)** late-arterial phase showed significant contrast agent retention.

**Figure 2:**
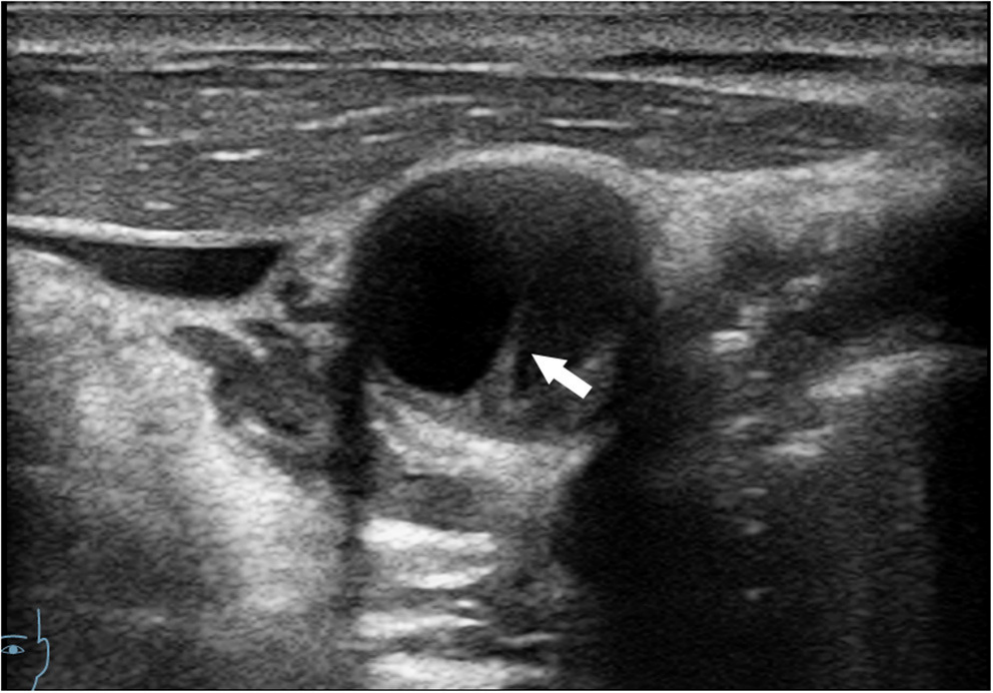
Conventional ultrasound showing axial view of CaW (arrow indicates shelf-like CaW).

The diagnosis of CaW was achieved with DSA, which was chosen for its high temporal and spatial resolution (20). DSA is considered the gold standard for CaW diagnosis (9). The CaW showed a contrast filling defect when assessed with DSA (21) (Figure 1). On conventional ultrasound, the CaW appears as a hypoechoic plaque with an axial shelf-like structure (16) (Figure 2). Upon assessment with CEUS, CaW showed delayed filling of contrast agent. Imaging examination results were read independently and blinded by two physicians in the department of neurology. If there were inconsistent judgments, they were determined after further discussion.

### Statistical Analysis

Statistical analysis was performed in SPSS 22.0 software (IBM SPSS Statistics 22.0). Categorical variables are presented as numbers (%). Continuous variables that were normally distributed were described as the mean ± standard deviation. Continuous variables that were not normally distributed were presented as the median and quartile intervals. The Chi-square test was used when comparing categorical variables. Continuous variables were compared using the *t*-test or Mann-Whitney *U*-test. *P* < 0.05 indicated that there was statistical difference.

## Results

A total of 17 CaW patients were enrolled in this study, including 4 females (23.5%) and 13 males (76.5%), with an average age of 59.41 (±10.86) years (Table 1). All 17 patients had unilateral CaW, including nine patients (52.9%) with left CaW and eight patients (47.1%) with right CaW. AIS occurred in 14 patients (82.4%). Among the 17 patients, 13 patients (76.5%) had hypertension, one patient (5.9%) had hyperlipidemia, eight patients (47.1%) had diabetes, two patients (11.8%) had coronary heart disease, and six patients had (35.3%) a history of stroke. Thrombosis occurred in five of 17 patients (29.4%). The median systolic blood pressure (SBP) value was 129 mmHg (IQR, 118–143 mmHg), the median diastolic blood pressure (DBP) value was 77 mmHg (IQR, 72–88 mmHg), and the median glucose value was 5.46 mmol/L (IQR, 4.89–6.08 mmol/L). The median TG value was 1.17 mmol/L (IQR, 0.73–1.94 mmol/L), the median TC value was 3.59 mmol/L (IQR, 2.91–4.88 mmol/L), the median HDL value was 1.12 mmol/L (IQR, 0.90–1.38 mmol/L), and the median LDL value was 2.06 mmol/L (IQR, 1.23–3.00 mmol/L).

**Table 1:** Clinical characteristics of CaW patients.

**Characteristics**	**All patients (*N* = 17)**
Age, yr, mean (±SD)	59.41 (±10.86)
Sex (male), *n* (%)	13 (76.5)
Hypertension, *n* (%)	13 (76.5)
Dyslipidemia, *n* (%)	1 (5.9)
Diabetes, *n* (%)	8 (47.1)
Coronary heart disease, *n* (%)	2 (11.8)
History of stroke, *n* (%)	6 (35.3)
SBP, mmHg, Median (IQR)	129 (118–143)
DBP, mmHg, Median (IQR)	77 (72–88)
Glucose, mmol/L, Median (IQR)	5.46 (4.89–6.08)
TG, mmol/L, Median (IQR)	1.17 (0.73–1.94)
TC, mmol/L, Median (IQR)	3.59 (2.91–4.88)
HDL, mmol/L, Median (IQR)	1.12 (0.90–1.38)
LDL, mmol/L, Median (IQR)	2.06 (1.23–3.00)
Stenosis (%), Median (IQR)	40 (30–50)
AIS, *n* (%)	14 (82.4)
Left CaW, *n* (%)	9 (52.9)
Thrombosis, *n* (%)	5 (29.4)
Smoking, *n* (%)	5 (29.4)
Alcohol, *n* (%)	3 (17.6)

*AIS, acute ischemic stroke; CaW, carotid web; DBP, diastolic blood pressure; HDL, high-density lipoprotein; LDL, low-density lipoprotein; TC, total cholesterol; TG, triglyceride; SBP, systolic blood pressure; yr, year*.

The clinical difference between the <60 years old CaW group and the ≥60 years old CaW group was compared in Table 2. The median admission glucose level in the <60 years old CaW group was found to be higher than the ≥60 years old CaW group [<60 years: 5.47 mmol/L (IQR, 4.88–6.69 mmol/L), ≥60 years: 5.39 mmol/L (IQR, 4.84–5.39 mmol/L)]. In addition, the median TC and LDL levels of patients in the <60 years old CaW group were higher than that of patients in the ≥60 years old CaW group [TC: <60 years 4.51 mmol/L (IQR, 2.91–5.47 mmol/L), ≥60 years old 3.28 mmol/L (IQR, 2.89–4.37 mmol/L); LDL: <60 years 2.84 mmol/L (IQR, 1.12–3.47 mmol/L), ≥60 years old 1.68 mmol/L (IQR, 1.28–2.27 mmol/L)]. Furthermore, there was a significant statistical difference about the thrombosis between the two groups [<60 years: 0 (0%), ≥60 years: 5 (62.5%), *P* = 0.005].

**Table 2:** Comparison of characteristics between the <60 years old CaW group and the ≥60 years old CaW group.

**Characteristics**	**Age <60 years (*N* = 9)**	**Age ≥60 years (*N* = 8)**	***P*-value**
Sex (male), *n* (%)	8 (88.9)	5 (62.5)	0.410
Hypertension, *n* (%)	6 (66.7)	7 (87.5)	0.312
Dyslipidemia, *n* (%)	1 (11.1)	0 (0)	0.331
Diabetes, *n* (%)	5 (55.6)	3 (37.5)	0.457
Coronary heart disease, *n* (%)	1 (11.1)	1 (12.5)	0.929
History of stroke, *n* (%)	3 (33.3)	3 (37.5)	0.858
SBP, mmHg, Median (IQR)	138 (120, 145)	126 (112, 138)	0.423
DBP, mmHg, Median (IQR)	77 (70, 88)	77 (71, 91)	1.000
Glucose, mmol/L, Median (IQR)	5.47 (4.88, 6.69)	5.39 (4.84, 5.39)	0.541
TG, mmol/L, Median (IQR)	1.17 (0.69, 2.44)	1.18 (0.81, 1.60)	0.606
TC, mmol/L, Median (IQR)	4.51 (2.91, 5.47)	3.28 (2.89, 4.37)	0.277
HDL, mmol/L, Median (IQR)	1.04 (0.83, 1.34)	1.24 (0.95, 1.41)	0.673
LDL, mmol/L, Median (IQR)	2.84 (1.12, 3.47)	1.68 (1.28, 2.27)	0.481
Stenosis (%), Median (IQR)	40.0 (30.0, 55.0)	35.0 (22.5, 47.5)	0.370
AIS, *n* (%)	7 (77.8)	7 (87.5)	0.600
Left CaW, *n* (%)	5 (55.6)	4 (50.0)	0.819
Thrombosis, *n* (%)	0 (0)	5 (62.5)	**0.005**
Smoking, *n* (%)	4 (44.4)	1 (12.5)	0.149
Alcohol, *n* (%)	3 (33.3)	0 (0)	0.072

*AIS, acute ischemic stroke; CaW, carotid web; DBP, diastolic blood pressure; HDL, high-density lipoprotein; LDL, low-density lipoprotein; TC, total cholesterol; TG, triglyceride; SBP, systolic blood pressure; yr, year; The bold values means p < 0.05*.

Seventeen patients received the corresponding treatment strategy after admission (Table 3), of whom seven patients (41.2%) received medical management, nine patients (52.9%) received carotid artery stenting (CAS), and one patient (5.9%) received carotid endarterectomy (CEA). Among patients who received medical management, there were five patients (71.4%) had right CaW, median length 7.1 mm (IQR, 4.5–13.0 mm), median width 2.5 mm (IQR, 1.6–2.9 mm), median stenosis rate 30% (IQR, 25–50%), and no recurrence at a median follow-up of 11 months (IQR, 4–13 months). Among patients who underwent CAS, seven patients (77.8%) had left CaW, with median length of 8.7 mm (IQR, 5.1–11.4 mm), a median width of 2.5 mm (IQR, 1.6–4.1 mm), a median stenosis rate of 40% (IQR, 30–55%), and no recurrence at a median follow up of 9 months (IQR, 6–20 months). Only 1 patient received CEA.

**Table 3:** Treatment of patients with CaW.

**Treatment**	***N***	**Age (yr), Median (IQR)**	**Sex (male), *n* (%)**	**Location (right),** ***n* (%)**	**Length (mm), Median (IQR)**	**Width (mm), Median (IQR)**	**Stenosis (%), Median (IQR)**	**Follow up (month), Median (IQR)**	**Recurrence, *n* (%)**
Medical management	7	58 (53–64)	4 (57.1)	5 (71.4)	7.1 (4.5–13.0)	2.5 (1.6–2.9)	30 (25–50)	11 (4–13)	0
CAS	9	63 (49–72)	8 (88.9)	2 (22.2)	8.7 (5.1–11.4)	2.5 (1.6–4.1)	40 (30–55)	9 (6–20)	0
CEA	1	58	1 (100)	1 (100)	8.9	5.1	50	2	0

*CAS, carotid artery stenting; CaW, carotid web; CEA, carotid endarterectomy; yr, year*.

**Figure 3:**
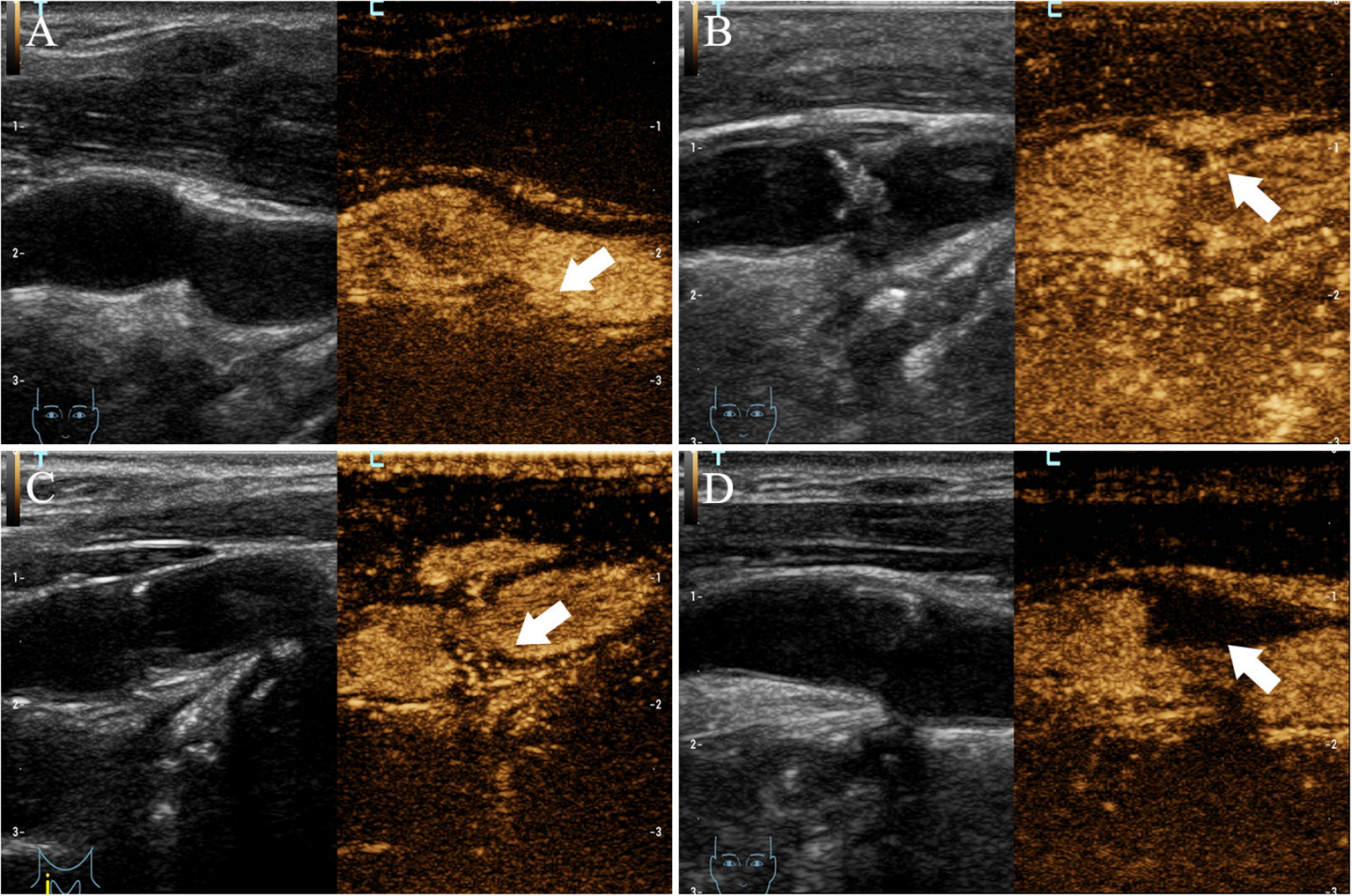
Imaging manifestations of CaW using CEUS. **(A–D)** all have obvious contrast filling defects (arrows indicate filling defects).

Upon conventional ultrasound, CaW appeared as a hypoechoic plaque that protrudes into the lumen. On CEUS, delayed infusion of the contrast agent was observed and a perfusion defect was revealed (Figure 3), where the web was located, to be protruding into the lumen after the injection of contrast agent. All 17 patients with CaW underwent conventional ultrasound and CEUS (Table 4). Among them, 16 patients (94.1%) with CaW were diagnosed by CEUS, and 1 patient was missed. Fourteen patients (82.4%) were diagnosed by conventional ultrasound, and three patients were missed. We found there was no statistical difference when comparing conventional ultrasound to CEUS in the diagnosis of CaW (*P* = 0.287). DSA confirmed thrombosis in five of 17 patients, five patients were diagnosed by CEUS (100%) and two patients were diagnosed by conventional ultrasound (40%), indicating that CEUS was statistically better than conventional ultrasound in the diagnosis of thrombosis (χ2 = 4.286, *P* = 0.038).

**Table 4:** Comparison of conventional ultrasound and CEUS in the diagnosis of CaW and thrombosis.

	**Conventional ultrasound**	**CEUS**	***χ2***	***P*-value**
	***n*(%)**	***n*(%)**		
CaW (*n* = 17)	14 (82.4)	16 (94.1)	1.133	0.287
Thrombosis (*n* = 5)	2 (40.0)	5 (100)	4.286	**0.038**

*CaW, carotid web; CEUS, contrast-enhanced ultrasound. The bold values means p < 0.05*.

## Discussion

The pathological characteristics of CaW are intimal thickening of the carotid artery and proliferation of fibroblasts (22). Angiography showed that most of the shelf-like structures associated with CaW were convex into the lumen (20). However, the exact etiology of CaW is still unclear and could involve factors such as genetics, presence of chronic vascular injury, hormone levels, and trophoblast vascular abnormalities (23). Our results found that hypertension accounted for a higher proportion of risk factors (76.5%), followed by diabetes (47.1%) and then smoking history (29.4%). Hypertension is associated with the onset of stroke and small-vessel disease (24). Therefore, it is very important for patients with CaW to control their blood pressure. In addition, fourteen patients (82.4%) experienced AIS. There was no significant difference when looking at the prevalence of the left CaW group and the right CaW group, which is consistent with previous study (25). Moreover, our results showed that there were statistical differences in the thrombosis between the <60 years old CaW group and the ≥60 years old CaW group (*P* = 0.005). However, there was no statistical difference in hematological indexes between the two groups. In this study, the proportion of male CaW patients was higher than that of female CaW patients, which contradicted the previous conclusion that there were more female patients than male patients (26, 27). This may be due to selection bias in the preponderance of male patients.

CaW leads to lumen stenosis and produces greater hemodynamic changes than atherosclerosis of a similar degree of lumen stenosis (10). The shelf-like structures of the CaW alter the distal hemodynamic pattern (22), resulting in the formation of a superimposed thrombus at the vortex (16). Superimposed thrombosis is associated with slow filling defects and turbulent blood flow (28). Thromboembolism can lead to stroke and neurological deterioration (29). Therefore, the examination of thrombosis is very important. Early detection of thrombi and taking corresponding treatment measures play an important role in saving patients' lives. In this study, the proportion of CaW patients with thrombosis reached 29.4%. Particularly, all patients with thrombosis were in the ≥60 years group, indicating that the incidence of CaW with thrombosis was higher in elderly patients. The risk of arterial and venous thrombosis increases with age (19, 30), which may be related to thrombin generation (31). The relationship and physiological mechanism between age and thrombosis in patients with CaW need to further study. Furthermore, the median stenosis rate of CaW patients was 40% (IQR, 30–50%). Patients with mild to moderate stenosis were at an increased risk of future vascular events (32).

There are many diagnostic methods for identifying CaWs. DSA has high temporal and spatial resolution, it can provide dynamic information on regional blood flow, which is the gold standard for CaW diagnosis (9). However, DSA is invasive and expensive (33, 34). Therefore, it is not suitable for routine examinations. CTA can quickly obtain high-resolution imaging and reconstructed-imaging in multiple planes (24). It can provide detailed vascular morphology information, and can distinguish between CaW, atherosclerosis, and artery dissection (35). However, CTA does not provide information on hemodynamics and lesion composition, and it exposes patients to radiation and iodine contrast agents (36). Conventional ultrasound is a non-invasive, convenient, and rapid examination method (37). Nevertheless, conventional ultrasound has limited depth and is highly dependent on doctors' subjectivity. CEUS was based on conventional ultrasound, and has more advantages (38). SonoVue was used as a contrast agent for CEUS, and there were no serious adverse reactions and no significant effects on liver and kidney function, so it was easily accepted by patients. Additionally, it can enhance the carotid lumen, allowing for a more in-depth assessment of arterial wall. CEUS can clearly show plaques in various parts of the vessel by signs of filling defects (39), provide higher image resolution and more quantitative data, such as quantitative plaque angiogenesis (13). Nevertheless, CEUS requires a high level of technical expertise from doctors because of the limited time-of-use of contrast agent. Conventional ultrasound and CEUS are important tools in the detection of carotid artery plaques, which indicates they have good diagnostic value for characterizing carotid artery plaques with cerebrovascular events (40). Some studies explored the diagnostic rate of atherosclerosis by CEUS and conventional ultrasound (41), and the results showed that CEUS could identify atherosclerotic plaques at a higher frequency (*P* = 0.02). Although CEUS does not show a high advantage in the diagnosis of CaW, it does show an advantage in the diagnosis of thrombus. The occurrence of AIS in CaW patients was associated with thrombus (34). Therefore, it is crucial to diagnose thrombosis timely for the treatment and medication of patients.

The treatment strategies for CaW mainly include medical management, CAS, and CEA. However, whether any of these treatment strategies are optimal remains unclear. Medical management alone has a higher recurrence rate (25). Although surgery is a first-line treatment strategy, patients may be at risk of vascular occlusion and more severe carotid stenosis (25, 42). CAS is the preferred treatment strategy because of its safety and effectiveness (43). Moreover, it has fewer perioperative complications (44). In this study, medical management, CAS, and CEA were used. The medical management in this study was antiplatelet therapy, including aspirin and cilostazol tablets. Antiplatelet therapy was chosen in most studies, and anticoagulant therapy was recommended by only few physicians (35). In a recent paper, Guglielmi et al. showed that 93% of ipsilateral CaW patients received drug therapy. Most of these patients were treated with antiplatelet therapy. However, the incidence of recurrent stroke was relatively higher in patients receiving anticoagulant therapy (27). Therefore, antiplatelet therapy is a reasonable treatment strategy in the patients with transient ischemic stroke or minor stroke (6). We suggested that antiplatelet therapy can be used in asymptomatic patients or patients with low stenosis rate of carotid artery, and surgical intervention is preferred for CaW patients with recurrent ischemic stroke. During the follow-up period, there was no recurrence of stroke in the CaW patients and the prognosis was good, indicating that these three methods are safe and effective. Due to the short follow-up period, long-term follow-up studies can be carried out in the future to explore the impact of different treatment strategies on the long-term prognosis of CaW patients.

CaW should be considered as a factor in patients with cryptogenic stroke. Doctors should improve their understanding of CaWs because CaW has a high risk of recurrence and early recognition and treatment are needed to help decrease the risk of additional strokes (3). This paper has several limitations. We only analyzed 17 CaW patients, which is a small sample size. The large sample size and multi-center studies are needed to verify our views in the future. The second limitation was the absence of histopathological verification. Finally, we did not compare the diagnostic rates of CaW between CTA and CEUS.

## Conclusions

CEUS may have a higher diagnostic accuracy for CaW with thrombosis, and it has a higher clinical application prospect for CaW patients.

## Data Availability Statement

The raw data supporting the conclusions of this article will be made available by the authors, without undue reservation.

## Ethics Statement

Ethical review and approval was not required for the study on human participants in accordance with the local legislation and institutional requirements. The patients/participants provided their written informed consent to participate in this study. Written informed consent was obtained from the individual(s) for the publication of any potentially identifiable images or data included in this article.

## Author Contributions

QZ and RL interpreted the data and drafted the manuscript. SF and FQ were responsible for the study coordination and implementation. WH and CT were responsible for design and organization. YZ acquired the data and designed the research. XL was responsible for funding and supervision. All authors contributed to the article and approved the submitted version.

## Funding

This work was supported by the National Natural Science Foundation of China (U20A20357), the Fundamental Research Funds for the Central Universities (WK9110000056), and by Program for Innovative Research Team of the First Affiliated Hospital of USTC.

## Conflict of Interest

The authors declare that the research was conducted in the absence of any commercial or financial relationships that could be construed as a potential conflict of interest.

## Publisher's Note

All claims expressed in this article are solely those of the authors and do not necessarily represent those of their affiliated organizations, or those of the publisher, the editors and the reviewers. Any product that may be evaluated in this article, or claim that may be made by its manufacturer, is not guaranteed or endorsed by the publisher.
